# Limited high-throughput screening compatibility of the phenuivirus cap-binding domain

**DOI:** 10.1038/s41598-023-50158-5

**Published:** 2023-12-20

**Authors:** Janna Scherf, Dominik Vogel, Sheraz Gul, Jeanette Reinshagen, Philip Gribbon, Maria Rosenthal

**Affiliations:** 1 Bernhard Nocht Institute for Tropical Medicine, Hamburg, Germany; 2 Fraunhofer Institute for Translational Medicine and Pharmacology (ITMP), Discovery Research ScreeningPort, Hamburg, Germany; 3 Fraunhofer Cluster of Excellence for Immune-Mediated Diseases (CIMD), Theodor Stern Kai 7, 60590 Frankfurt, Germany; 4 Center for Structural Systems Biology, Hamburg, Germany

**Keywords:** RNA-binding proteins, Viral infection, Drug screening, Target identification

## Abstract

Bunyaviruses constitute a large and diverse group of viruses encompassing many emerging pathogens, such as Rift Valley fever virus (family *Phenuiviridae*), with public and veterinary health relevance but with very limited medical countermeasures are available. For the development of antiviral strategies, the identification and validation of virus-specific targets would be of high value. The cap-snatching mechanism is an essential process in the life cycle of bunyaviruses to produce capped mRNAs, which are then recognized and translated into viral proteins by the host cell translation machinery. Cap-snatching involves cap-binding as well as endonuclease functions and both activities have been demonstrated to be druggable in related influenza viruses. Here, we explore the suitability of the phenuivirus cap-binding function as a target in medium- and high-throughput drug discovery approaches. We developed a range of in vitro assays aiming to detect the interaction between the cap-binding domain (CBD) and the analogue of its natural cap-ligand m^7^GTP. However, constricted by its shallow binding pocket and low affinity for m^7^GTP, we conclude that the CBD has limited small molecule targeting potential using classical in vitro drug discovery approaches.

## Introduction

Severe fever with thrombocytopenia syndrome virus (SFTSV) and Rift Valley fever virus (RVFV) are segmented single-stranded RNA viruses belonging to the *Phenuiviridae* family within the order *Bunyavirales* (https://talk.ictvonline.org/taxonomy/). Bunyaviruses can infect a range of mammalian, invertebrate and plant hosts, including humans^1,2^. While SFTSV is prevalent in East Asian countries^3^, RVFV is endemic to sub-Saharan Africa and the Arabian Peninsula^4^. Until now, no antivirals or other effective therapeutic approaches are available to treat bunyaviral infections. Therefore, the WHO R&D Blueprint emphasizes the importance to develop medical countermeasures against several bunyaviruses, as they are a threat to global health and the economy especially in Low- and Middle-income countries^5^.

Bunyavirus genome replication and transcription proceeds in the cell cytoplasm and is mediated by the viral 250–450 kDa large (L) protein, which contains RNA-dependent RNA polymerase (RdRp), endonuclease and cap-binding functions^6,7^. While genome replication yields identical genome copies by de novo RNA synthesis, transcription produces capped viral mRNA^8^. Bunyaviruses lack enzymes to produce a 5′ RNA cap structure and thus employ a so-called cap-snatching mechanism mediated by the cap-binding and endonuclease activities of the L protein, which bind to and cleave off capped RNA fragments of host mRNA. Subsequently, these capped RNA fragments are used to prime viral transcription and capped viral mRNAs are recognized and translated into proteins by the cellular translation machinery^8,9^.

For SFTSV and RVFV, the cap-binding domain (CBD) has been shown to be located in the C-terminal region of the L protein and residues interacting with the cap-analogue m^7^GTP in the crystal structures have been proven to be essential for viral transcription in cells^7,10^. The crystal structures of these CBDs in complex with m^7^GTP were determined and have overall structural similarity to the influenza virus cap-binding domain, but differ slightly from influenza virus CBD in the mode of cap-recognition^7,8,10^. All three viral CBDs, similarly to cellular and other viral cap-binding proteins such as eIF4E, nuclear cap-binding complex (CBC) and vaccinia virus VP39, sandwich m^7^GTP between two aromatic sidechains and specifically select for the methylated over the non-methylated GTP^11–13^. The specific but relatively low-affinity interaction of SFTSV and RVFV CBD with an m^7^GTP ligand was demonstrated previously using isothermal titration calorimetry (ITC)^7,10^.

To date, several small molecules which target the influenza virus CBD to block cap binding and thereby inhibit viral transcription have been identified, including pimodivir^14^ and roscovitine^15^. While pimodivir was terminated in phase III clinical trials because of the development of viral resistance^16^ and no significant benefit over the standard of care against influenza virus infection^17^, roscovitine already entered several phase I and II clinical trials for cancer therapy^18,19^ and is now being explored as a possible influenza virus infection treatment^20^. As bunyaviral CBDs and influenza virus CBD share structural features and functional roles, transferring this principle of cap-binding inhibition to bunyaviruses might open up new perspectives for antiviral treatment. As cap-snatching is a key process in the bunyavirus life cycle, the cap-binding function could be a valuable, potentially broad-spectrum drug targeting mechanism.

Using a panel of in vitro assays to perform medium-throughput screening (MTS) of small-molecule libraries, we attempted to validate SFTSV CBD as an antiviral target. Fluorescence polarization (FP) and surface plasmon resonance (SPR) assays were developed to identify SFTSV CBD-specific ligands. Additionally, a microscale thermophoresis (MST) assay was established for assessment of compound–target engagement. The relatively weak interaction between the CBD and the cap-analogue m^7^GTP in vitro prevented the development of robust screening assays based on competition-based FP or SPR readouts. This is consistent with the m^7^GTP binding cavity being relatively shallow in depth. We therefore conclude that the SFTSV CBD displays limited tractability using classical small molecule in vitro drug discovery approaches.

## Results

### Protein purification and thermal stability assays

Only the C-terminal part of the SFTSV L protein, the cap-binding domain (CBD), was used for assay development. Depending on the assay format, the CBD was produced without a tag or with an N-terminal histidine-, cysteine- or Strep-tag. Proteins were purified as described in the Methods. Protein monodispersity and purity were confirmed by size exclusion chromatography and SDS-PAGE (Supplementary Fig. 1). Moreover, thermal stability assays (TSAs) were performed as an additional quality control, to verify protein stability (Supplementary Fig. 2a,b). It has been shown previously, that SFTSV and RVFV CBD display a higher thermal stability in the presence of m^7^GTP but not GTP indicating specific binding of the m^7^GTP cap-analogue to the CBD^7,10^. We therefore tested the thermal stability of the SFTSV and RVFV CBD in presence of the influenza virus cap-binding inhibitor pimodivir^14^ and, interestingly, observed a destabilization of the protein instead of a stabilization (Supplementary Fig. 2b). A similar effect has been observed with RVFV CBD in the presence of ATP^7^. This destabilization may be due to the ligand pushing into the hinge connecting the β-hairpin with the central β-sheet of the CBD, thereby rather forcing the m^7^GTP binding site to open up instead of stabilizing a closed conformation of the domain by the interaction with a ligand. To confirm this hypothesis by structural data, we therefore also attempted to co-crystallize the SFTSV and RVFV CBDs with the inhibitor pimodivir^14^ as well as soaking of protein crystals with the inhibitor, but without success. Therefore, this proposition still requires confirmation by future studies. While a thermal stabilization is generally accepted to indicate binding of a ligand, a destabilizing effect is difficult to interpret as the origin of changes may well arise from multiple underlying influences on structural stability related to the physico-chemical properties of both the ligand and the protein of interest, pH of the environment, as well as the level and type of ions etc. In summary, the interpretation of the thermal stability assay is difficult. Moreover, as this assay consumes a relatively high amount of protein and compound, it was not considered a viable option for MTS but rather as a possible counter assay to characterize hits identified from high throughput assays. We do not exclude fragment screening as a viable option for drug discovery targeting the CBD, though.

### Establishment of a fluorescence polarization (FP) assay

**Figure 1:**
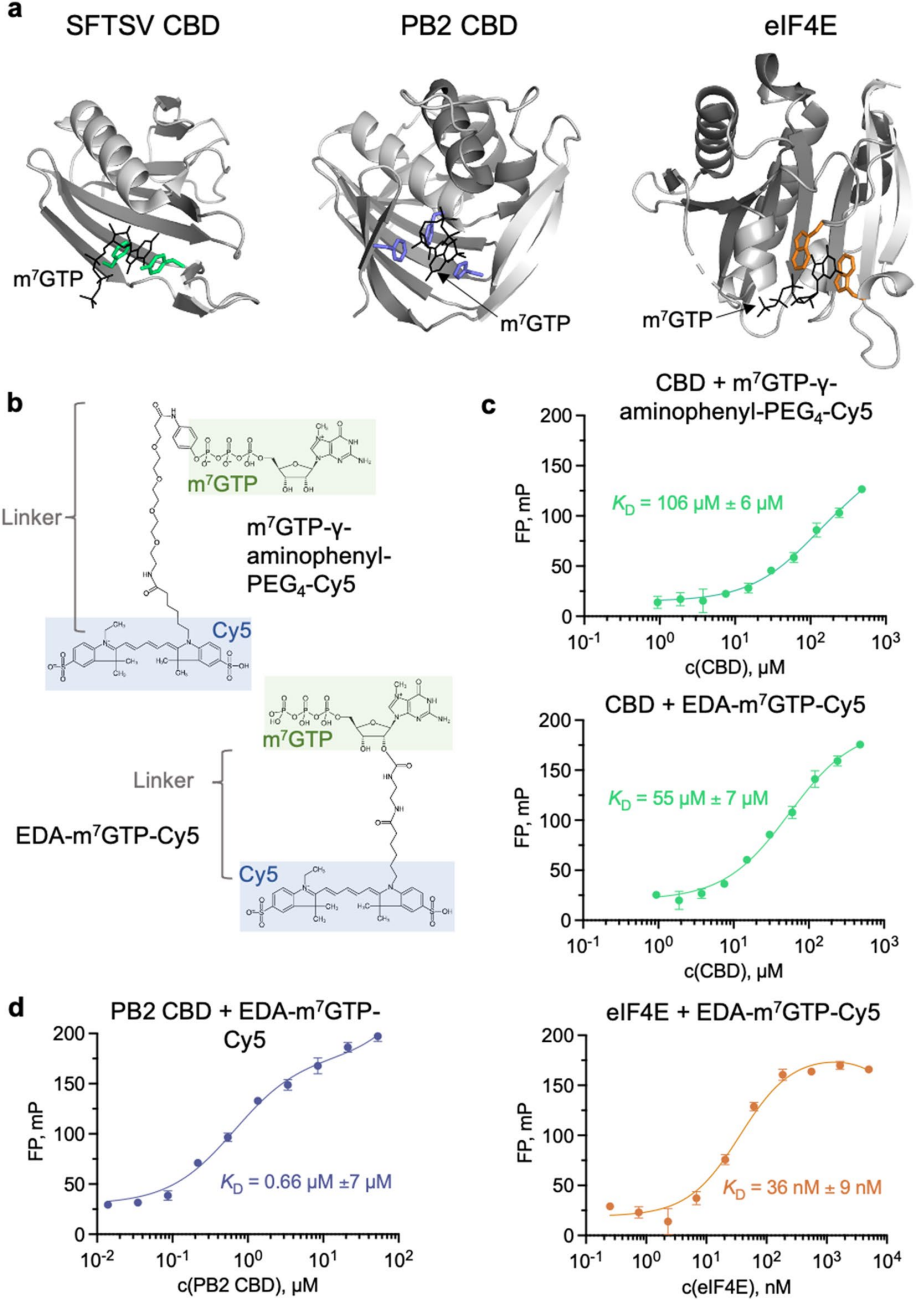
Fluorescence polarization experiments. (**a**) The figure shows crystal structures of SFTSV CBD (6XYA), PB2 CBD (2VQZ) and eIF4E (1L8B) in complex with m^7^GTP (presented as lines in black). Proteins are displayed as ribbon diagram with the side chains involved in the m^7^GTP interaction shown as sticks. The figure was created with PyMOL 2.5. (**b**) Structural formulas of the employed tracers: m^7^GTP-γ-aminophenyl-PEG_4_-Cy5 and EDA-m^7^GTP-Cy5 (Jena Bioscience GmbH). (**c**) Detection of the interaction of SFTSV CBD with the different tracers (upper panel: m^7^GTP-γ-aminophenyl-PEG_4_-Cy5, lower panel: EDA-m^7^GTP-Cy5) and (**d**) of the interaction of PB2 CBD and eIF4E with EDA-m^7^GTP-Cy5 via FP. The FP signal was measured in presence of 5 nM tracer and the results were fitted according to a single site binding model. Measurements were done in technical triplicates and are shown as representatives of at least 3 biological replicates (all biological replicates are shown in Supplementary Fig. 3). Fitting and plotting was done in GraphPad Prism 9.5.

Crystal structures of SFTSV and RVFV CBDs in complex with the cap-analogue m^7^GTP revealed that the ligand is stacked via its base between the two aromatic residues F1703 and Y1719 of SFTSV CBD and F1713 and Y1728 of RVFV CBD, respectively^7,10^ (Fig. 1a). We aimed at developing a competitive FP assay amenable to MTS, in which potential hit compounds could displace fluorescently labelled m^7^GTP (tracer) causing a drop in the FP signal. In order to find a fluorescently labelled cap-analogue serving as a tracer, which should not interfere with binding to the CBDs, m^7^GTP-γ-aminophenyl-PEG4-Cy5 and EDA-m^7^GTP-Cy5 (Fig. 1b) were tested. In m^7^GTP-γ-aminophenyl-PEG4-Cy5, a linker connects the Cy5 label to the γ-phosphate of m^7^GTP, keeping it distant from the base. To examine if the CBD binds to fluorescently labelled m^7^GTP, a binding curve was obtained by titration of the tracer with increasing SFTSV CBD (without a tag) concentrations up to 481 µM. Although an increase in the FP signal was detected, no saturation was reached (Fig. 1c, upper panel). We suspected that the so-called propeller effect could have an effect on the FP readout^21^. This effect can be caused by a too long linker allowing the Cy5 label to rotate to a certain extent in solution while the tracer remains bound to the protein. Repetition of the experiment with EDA-m^7^GTP-Cy5, labelled at the ribose with a shorter linker, resulted in a marked increase of the FP signal at lower tracer concentrations, indicating improved binding (Fig. 1c, lower panel). Fitting the data to a single site binding model using GraphPad Prism 9.5 resulted in a *K*_D_ of 55 µM ± 7 µM, which is in a similar range as the previously determined *K*_D_ of 138 µM ± 17 µM by ITC for SFTSV CBD and unlabeled m^7^GTP^10^. However, saturation was not reached, which significantly limits the reliability of the fit. To confirm the assay performance, we used influenza virus PB2 CBD and human eIF4E as positive controls, since they both exhibit a similar binding mode by stacking m^7^GTP via an aromatic sandwich (Fig. 1a). The determined *K*_D_ values of PB2 and eIF4E resembled those reported previously (Fig. 1d)^22,23^. Overall, these data suggest a low affinity of SFTSV CBD for m^7^GTP, leading to the requirement of high protein concentration to ensure an adequate assay window. This renders the establishment of a competitive FP format assay not tractable for undertaking larger scale screening campaigns.

### Evaluation of surface plasmon resonance (SPR) assay

**Figure 2:**
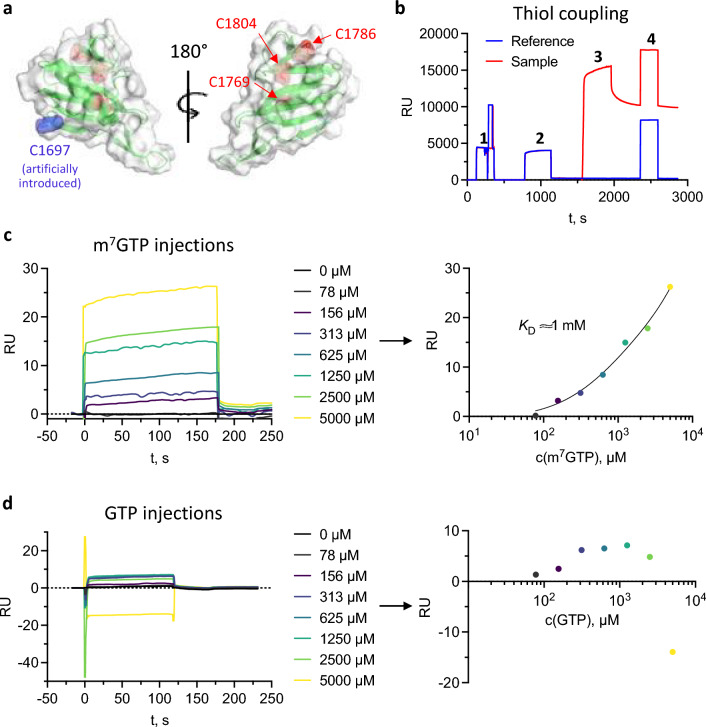
Surface plasmon resonance experiments. (**a**) Crystal structures of SFTSV CBD presented as ribbon diagram with the proteins surface in light grey, the intrinsic cysteines in red and the introduced N-terminal cysteine in blue. The figure was created with PyMOL 2.5. (**b**) Immobilization of SFTSV CBD Cys via ligand thiol coupling on an amine sensorchip: 1. activation with NHS/EDC, 2. introduction of reactive disulfide groups, 3. injection of SFTSV CBD Cys, 4. deactivation of unreacted disulfide groups. (**c**) Injection of increasing m^7^GTP concentrations and (**d**) of increasing GTP concentrations on the immobilized SFTSV CBD Cys (left side). The right side shows the equilibrium analysis of the data (Analyzer 3 Software, Bruker Daltonics GmbH). Plots were created in GraphPad Prism 9.5.

In order to overcome the need for high protein concentrations in FP based assays, SPR was considered as alternative potential MTS assay modality. Here, immobilized CBD on a sensor chip was reused for multiple measurements reducing the total amount of protein required. To this end, different protein immobilization methods were tested: amine coupling, Strep-tag:NeutrAvidin interaction and thiol coupling. Amine coupling constitutes a simple, well-established method for immobilization and worked well for the SFTSV CBD (without a tag), but the following m^7^GTP injections produced no dose-dependent signal (Supplementary Fig. 4b). Although there are several lysine residues present on the CBD surface, immobilization in the correct orientation ensuring accessibility and functionality of the cap-binding site could be restricted. In particular, four lysine residues (Lys1705, Lys1708, Lys1720 and Lys1722) are located in the β-hairpin and the β-hairpin linker region (Supplementary Fig. 4a). Covalent immobilization via these side chains likely restricts movement of the β-hairpin relative to the adjacent β-sheet, which is expected to be important for m^7^GTP binding. Unspecific interaction of the ligand or/and the analyte with the underlying chip surface is also possible and could be problematic in SPR. As an alternative, immobilization of the N-terminal strep-tagged SFTSV CBD to a neutravidin coated chip was performed. This interaction was expected to be more specific and ensure beneficial orientation of the CBD as the Strep-tag is located opposite of the cap-binding site. However, this approach did not result in sufficient amounts of immobilized CBD and subsequent m^7^GTP injections caused no dose-dependent signal changes (Supplementary Fig. 4c). The best results for protein coupling and ligand interaction were achieved using thiol coupling. As the intrinsic cysteine residues of SFTSV CBD are all beneath the proteins surface, an N-terminal cysteine was introduced (Fig. 2a). After immobilization (Fig. 2b), m^7^GTP injections up to 5 mM resulted in a dose-dependent signal increase (Fig. 2c). However, saturation of the immobilized protein was not reached, significantly limiting the reliability of the applied fit. Moreover, the estimated *K*_D_ of approximately 1 mM was significantly higher compared to previous FP and ITC derived results^7,10^. Injecting GTP as a negative control on the thiol-coupled SFTSV CBD did not result in a dose-dependent signal increase (Fig. 2d). Nevertheless, there is no reliable positive binding control available at the moment, to verify that the interaction of the SFTSV CBD with the cap-analogue m^7^GTP is detectable with SPR. Furthermore, reproduction of the results along with reusage and regeneration of the chip, with DTT treatment, failed. The ineffective regeneration of the chip might indicate unspecific interaction of the CBD with or even aggregation on the sensor chips surface. This would substantially reduce the protein’s biological function and hence, the ability to specifically interact with m^7^GTP, which could explain the comparably low affinity for m^7^GTP that was measured. In summary, we were unable to establish a reliable SPR-based assay to detect the interaction of the SFTSV CBD with small molecule ligands.

### Evaluation of microscale thermophoresis (MST) as an alternative assay

**Figure 3:**
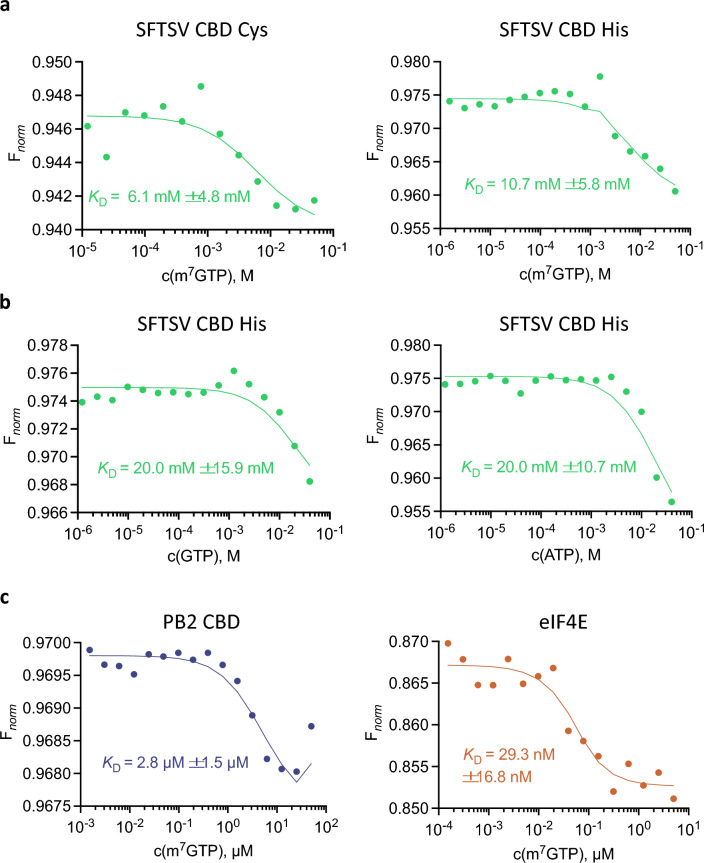
Microscale thermophoresis experiments. Normalized MST binding curves of labelled proteins titrated with m^7^GTP, GTP or ATP. (**a**) 100 nM SFTSV CBD Cys (left) and 100 nM SFTSV CBD His (right) are titrated with m^7^GTP. (**b**) 100 nM SFTSV CBD His was titrated with GTP (left) and ATP (right). (**c**) The control proteins PB2 (50 nM, left) and eIF4E (50 nM, right) were titrated with m^7^GTP. The analysis was done with ThermoAffinity (spc.embl-hamburg.de)^25^ and plots were created in GraphPad Prism 9.5.

MST was not considered as an MTS assay due to its limited throughput, but rather a possible confirmatory assay as part of profiling potential hits emerging from FP or SPR high throughput assays. Initially, investigation on whether the interaction of the CBD with a fluorescently labelled EDA-m^7^GTP-Cy5 can be detected was undertaken. However, upon titration of EDA-m^7^GTP-Cy5 with increasing SFTSV CBD (without a tag) concentrations, the absolute fluorescence intensity increased as well. The model used for fitting the MST signal assumes identical weighting of the bound and unbound states, which is not applicable if bound and unbound states differ in absolute fluorescence^24^. For a fluorescence change that is larger than 20%, the manufacturer (Nanotemper) recommends analyzing the initial fluorescence signal instead of the MST signal. However, it is unknown if the fluorescence change is solely caused by interaction of the CBD with m^7^GTP or whether other effects are also involved. Therefore, utilizing the absolute fluorescence intensity method to determine binding kinetics was not performed. Subsequently, attempts to titrate fluorescently labelled protein with m^7^GTP were performed. For protein labelling, N-terminally cysteine-tagged or N-terminally His-tagged SFTSV CBDs were coupled with RED-MALEIMIDE 2nd Generation dye and RED-Tris NTA 2nd Generation dye (Nanotemper), respectively. Titration of the differently labelled CBDs with m^7^GTP (up to 50 mM) produced similar curves with an initial slight increase of the *F*_norm_, followed by a stronger decrease from 500 µM upwards (Fig. 3a). Binding curve data fitting resulted in *K*_D_ values of approximately 6 mM in both setups, which is higher than observed in previous experiments. As similar *K*_D_ values were obtained upon titrating SFTSV CBD with GTP and ATP (Fig. 3b), the decreasing *F*_norm_ is most likely not due to specific interactions, limiting the accurate interpretation of the results. Control experiments with eIF4E and PB2 CBD resulted in *K*_D_ values similar to those reported previously^22,23^, indicating that in general the underlying assay principle is biologically relevant (Fig. 3c). However, in conclusion, MST does not appear to be a suitable method to detect the rather weak interaction between SFTSV CBD and m^7^GTP.

## Discussion

The cap-snatching mechanism is essential and specific to segmented negative-strand RNA viruses involving both a cap-binding and an endonuclease function of the viral polymerase proteins. While influenza viruses perform cap snatching in the nucleus, bunyaviruses accomplish this process in the cytoplasm. It has been shown that the cap-binding domains of influenza viruses and bunyaviruses share structural and functional homology, although, the exact mode of cap-recognition differs between the two^7^. For example, the CBD of influenza virus PB2 protein has several more contacts to the capped RNA than observed in SFTSV and RVFV CBDs^8^. The CBD of La Crosse virus (*Peribunyaviridae* family) does not even employ an aromatic sandwich for stacking but an aromatic and an arginine sidechain^26^. It was also shown that the influenza virus polymerase complex interacts with more residues of the capped RNA in addition to the m^7^GTP, specifically with bases 1–5 following the 5′ cap^27^. Also for La Crosse virus L, interaction of the L protein with several nucleotides downstream of the cap structure has been shown^26^. Recent structural data on SFTSV full-length L protein during viral transcription demonstrate a recognition of the first four bases after the cap, arising from electrostatic interactions with the phosphate backbone as well as sequence-independent base stacking^28^. These additional interactions will contribute to a higher affinity for capped RNA but the contribution of the CBD to the overall energy of that interaction is poorly understood. However, it seems clear that the CBD determines the specificity for m^7^GTP over GTP but also the 5′-5′ triphosphate linkage of the cap to the RNA likely is an important factor in selecting capped RNA over “ordinary” RNA with a terminal GTP. Additionally, host cell capped RNA is usually also 2′-O-methylated at the first ribose (cap1) and sometimes also at the second ribose (cap2) following the 5′ cap, which likely also contributes to the specific recognition of capped RNA. For these reasons, using the CBD as a minimal system for small-molecule screening approaches may have considerable limitations. However, using the full-length L protein with its several distinct RNA binding sites^29,30^ likely comes with problems of binding specificity. The tested assay systems and setups did all not allow for MTS or HTS screening. While on the one hand, the relatively low affinity of the CBD for the cap-analogue m^7^GTP (cap0) in principle constitutes an opportunity for small molecules to bind more efficiently to the CBD than the cap itself, this low affinity still posed a major problem for the assay development. Although this was not unexpected considering previous publications, it was different from other cap-binding proteins we used as positive controls and the biological relevance of this observation is unclear^8^. In addition, although we did not manage to obtain co-crystals with the CBD and pimodivir, we do not exclude that fragment screening may be a suitable method for drug discovery in this case. As a more targeted strategy, specific nucleoside analogues could be tested in lower-throughput assays.

In conclusion, the SFTSV CBD displays limited tractability towards existing approaches based on small molecules and therefore has restricted small molecule targeting using classical in vitro drug discovery approaches.

## Methods

### Cloning, expression and purification of SFTSV CBD mutants

The L gene region corresponding to amino acid residues 1695–1810 of SFTSV strain AH12 (GenBank accession no. HQ116417) was cloned into a pOPINF vector using the NEBuilder HiFi DNA Assembly Cloning Kit (New England BioLabs). The protein, referred to as SFTSV CBD, was expressed (i) with an N-terminal 6xHistidine-tag, (ii) an N-terminal 6xHistidine-tag plus an additional cysteine residue or (iii) an N-terminal 6xHistidine and Strep tandem-tag in *E. coli* strain BL21 Gold (DE3) (Novagen) at 17 °C overnight using TB medium and 0.5 mM isopropyl-β-D-thiogalactopyranosid for induction. After pelleting, the cells were resuspended in 50 mM Na-phosphate pH 7.5, 150 mM NaCl, 10 mM imidazole, Complete protease inhibitor EDTA-free (Roche), 0.4% (v/v) Triton X-100 and 0.2 mg/mL lysozyme and subsequently disrupted by sonication. After centrifugation, the protein was purified from the soluble fraction by Ni affinity chromatography. Buffers containing either 50 mM imidazole and 1 M NaCl or 50 mM imidazole and 150 mM NaCl were used for the washing steps. Depending on the requirements of the assay setup, the 6xHistidine-tag was either cleaved on column by a GST-tagged 3C-Protease (30 min incubation at room temperature) or the tagged protein was eluted with 300 mM imidazole. The additional cysteine residue and the strep tag, respectively, were not removed. Afterwards, the protein was concentrated and submitted to size exclusion chromatography (Superdex 200, buffer: 50 mM Na-phosphate pH 6.5, 150 mM NaCl, 10% (w/v) glycerol). Purified proteins were concentrated using a centrifugal device, flash frozen in liquid nitrogen, and stored at – 80 °C until further use.

### Thermal stability assay (TSA)

Thermal stability of proteins was measured by thermofluor assay^31^. Final protein concentrations were 8 µM (SFTSV CBD, RVFV CBD) and 10 µM (PB2) in 50 mM Na-phosphate pH 6.5, 150 mM NaCl, 10% (w/v) glycerol and Sypro-Orange (final dilution 1:1000). The assay was performed with either no additive or between 0.25 and 10 mM m^7^GTP or pimodivir from a 10 × stock. Controls included only DMSO. Melting curves were recorded from 20 to 84 °C and melting points at 50% unfolding were extracted from the normalized melting curves.

### Fluorescence polarization (FP) assay

The FP assay was performed in 384-well plates (Greiner Art. 784900) using Cy5 labelled m^7^GTP (m^7^GTP-γ-aminophenyl-PEG4-Cy5 and EDA-m^7^GTP-Cy5, Jena Bioscience) as detection probe (tracer). The tracer was diluted to 20 nM in 10 mM Na-phosphate pH 7.0 and 0.1% CHAPS (v/v). Serial dilutions of SFTSV CBD, PB2 and eIF4E were done in 10 mM Na-phosphate pH 7.0, 300 mM NaCl and 0.1% CHAPS (v/v). Subsequently, 5 µL tracer and 5 µL per protein dilution were mixed in one well of the assay plate, so that the final assay conditions were 10 mM Na-phosphate pH 7.0, 150 mM NaCl, 0.1% CHAPS (v/v), 10 nM tracer and varying protein concentrations. After incubation for 1 h at room temperature, FP measurement was carried out with an EnVision Multilabel Reader 2103 (PerkinElmer Inc.) at an excitation wavelength of 620 nm and recording emission at a wavelength of 688 nm. Results were analyzed with the software GraphPad Prism (Version 9.5 for Mac OS, GraphPad Software, Boston, Massachusetts USA, www.graphpad.com).

### Surface plasmon resonance (SPR) assay

SPR was conducted with a SIERRA SPR-16 instrument (Bruker Daltonics GmbH). Amine coupling was performed with a High Capacity Amine Sensor (Bruker Daltonics GmbH). Activation of the sensor surface with 100 mM N-Hydroxysuccinimide (NHS) and 100 mM 1-Ethyl-3-(3-dimethylaminopropyl)carbodiimid (EDC) in 50 mM MES pH 5.0 was followed by immobilization of the SFTSV CBD. The protein was injected at a concentration of 100 µg/mL and a flow rate of 5 µL/min in SPR running buffer (50 mM NaP pH 6.5, 10 mM NaCl, 0.05% Tween 20 (v/v)). Unreacted NHS-esters were subsequently deactivated by injecting 1 M ethanolamine hydrochloride pH 8.5 at a flow rate of 10 µL/min.

For Strep-tag:NeutrAvidin coupling the surface of a Biotin-Tag Capture Sensor (Bruker Daltonics GmbH) was prepared via three injections of 1 M NaCl, 10 mM NaOH (Bruker Daltonics GmbH). Subsequently, 200 nM SFTSV CBD containing a strep-tag in SPR running buffer was injected at a flow rate of 5 µL/min.

SFTSV CBD with an additional cysteine was immobilized on a High Capacity Amine Sensor (Bruker Daltonics GmbH) by ligand thiol coupling. After activation of the sensor surface by injecting 100 mM NHS and 100 mM EDC in 50 mM MES pH 5.0 (XanTec bioanalytics GmbH), reactive disulfide groups are introduced by the injection of 80 mM PDEA thiol coupling reagent in 0.1 M sodium borate pH 8.5 (XanTec bioanalytics GmbH). Next, 100 µg/mL SFTSV CBD with an additional cysteine diluted in 2 mM sodium acetate pH 5.0 (Bruker Daltonics GmbH) was immobilized at a flow rate of 5 µL/min, followed by deactivation of unreacted disulfide groups with 50 mM L-cysteine in 1 M NaCl and 100 mM sodium acetate pH 4.0 (XanTec bioanalytics GmbH). For binding experiments, m^7^GTP and GTP were injected in SPR running buffer at a flow rate of 10 µL/min. SPR data were analyzed using the Analyzer 3 software (Bruker Daltonics GmbH).

### Microscale thermophoresis (MST) assay

MST experiments were performed with a Monolith NT.115 (Nanotemper). His-tagged proteins were labelled with RED-MALEIMIDE 2nd Generation dye and SFTSV CBD with an additional cysteine residue was labelled with RED-Tris NTA 2nd Generation dye according to the protocols provided by Nanotemper. Dilutions of the interaction partners were prepared in 50 mM Na-phosphate pH 6.5, 150 mM NaCl and 0.05% Tween-20 (v/v). Protein was added in a 1:1 ratio to serial dilutions of m^7^GTP, GTP or ATP, so that the assay was conducted at final protein concentrations of 100 nM SFTSV CBD, 50 nM PB2 and 50 nM eIF4E in premium grade capillaries (Nanotemper). After incubation for 30 min at room temperature, measurements were performed at medium MST-power and 20% excitation power. The resulting time courses of fluorescence intensities were analyzed using the online data-analysis ThermoAffinity platform^25,31^.

### Supplementary Information

Supplementary Figures.

## Data Availability

Gene sequences are available from GenBank for SFTSV strain AH12 (GenBank accession no. HQ116417, https://www.ncbi.nlm.nih.gov/nuccore/HQ116417). Uncropped gel images are provided in Supplementary Fig. 5. Raw data are available from the corresponding authors upon reasonable request.
