# Supplementary material for: Limited high-throughput screening compatibility of the phenuivirus cap-binding domain
Source: Sci Rep. 2023 Dec 20;13:22820. doi: 10.1038/s41598-023-50158-5 (PMC10739838; doi:10.1038/s41598-023-50158-5)
Supplement: Supplementary file 1 — Supplementary Figures. [file 41598_2023_50158_MOESM1_ESM.pdf]

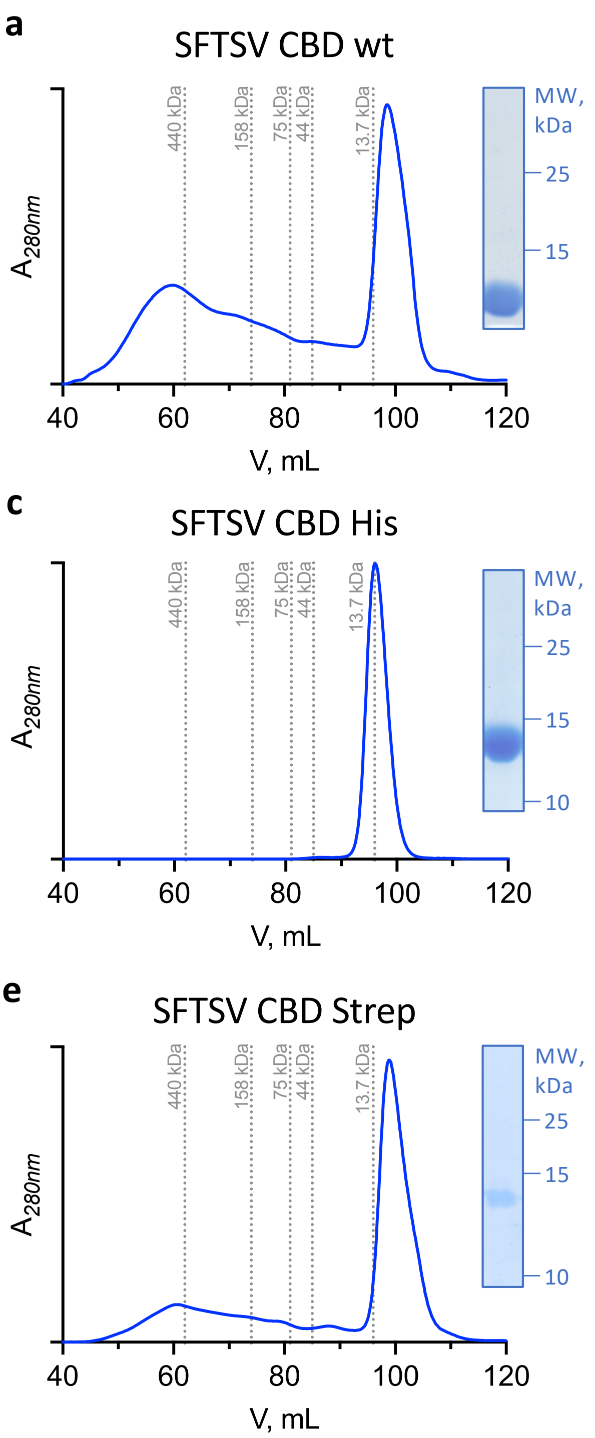

Supplementary Figure 1: Size exclusion chromatogray and SDS PAGE of CBD mutants. Size exclusion chromatography was performed with a Superdex 200 column and protein containing fractions were analyzed via 15% polyacrylamide gels stained with coomassie. All mutants are highly pure and monodisperse. **a)** Wilde type SFTSV CBD. **b)** Wilde type RVFV CBD. **c)** 6xHis tagged SFTSV CBD. **d)** Cys tagged SFTSV CBD. **E)** Strep tagged SFTSV CBD. Plots were created in GraphPad Prism 9.5.

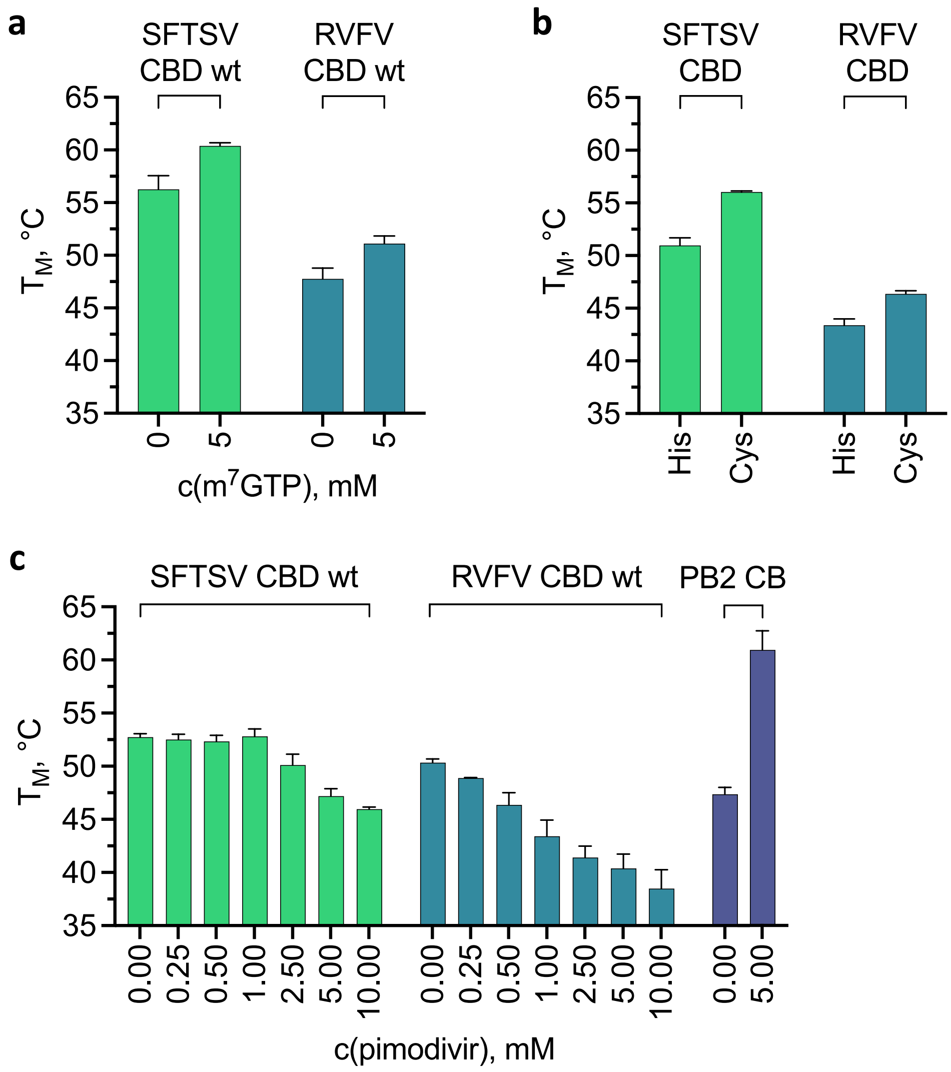

Supplementary Figure 2: Thermal stability assays. The thermal stability was measured of **a)** SFTSV CBD wt and RVFV CBD wt in presence of 0 and 5 mM of the cap-analogue m^7GTP, **b)** the different CBD mutants SFTSV CBD His, SFTSV CBD Cys, RVFV CBD His and RVFV CBD Cys and **c)** SFTSV CBD wt, RVFV CBD wt and PB2 CBD in presence of increasing pimodivir concentrations. Melting temperatures are presented as mean and standard deviations of three independent measurements. Plots were created in GraphPad Prism 9.5.

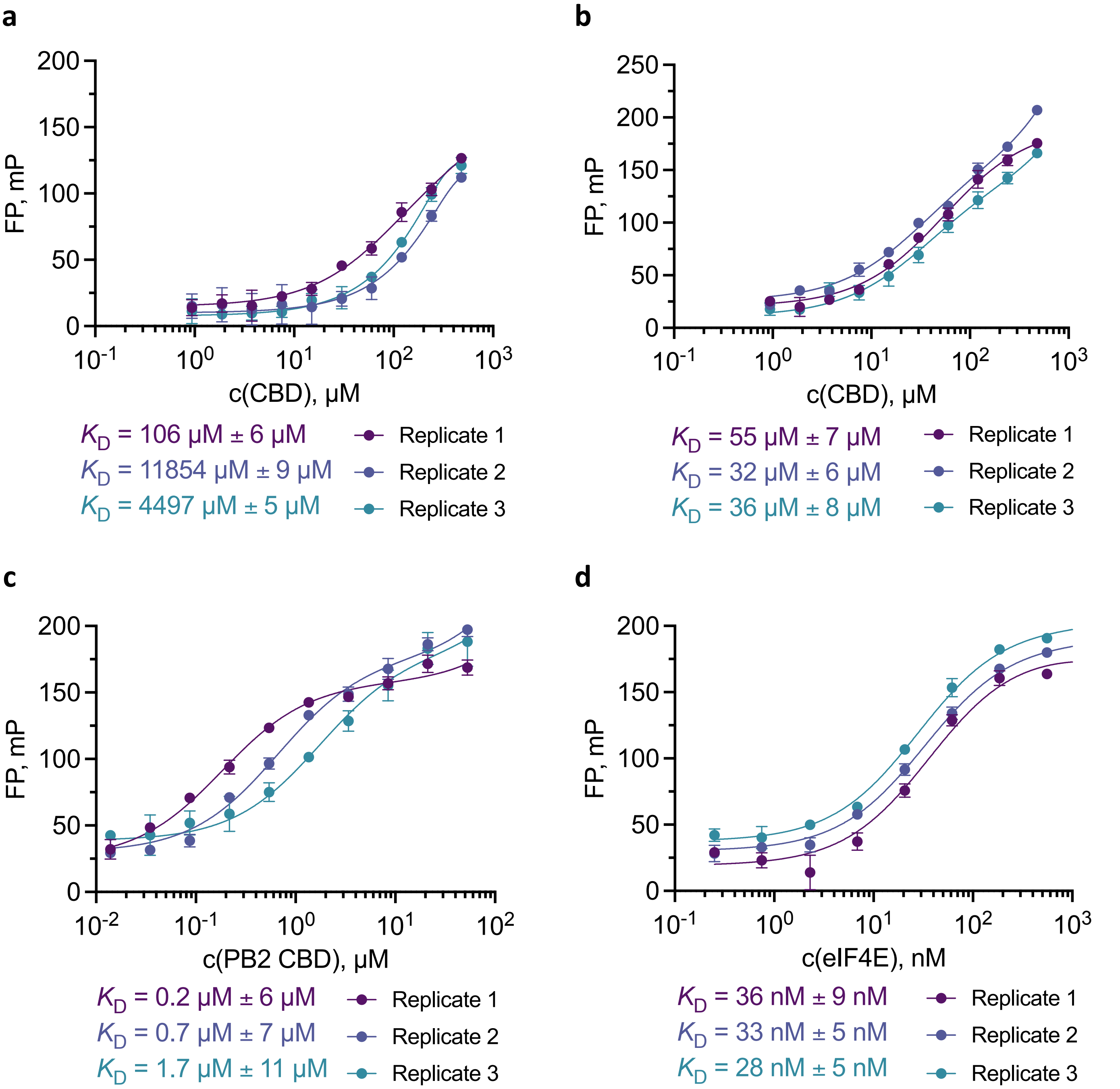

Supplementary Figure 3: Biological replicates of FP experiments. Detection of the interaction of SFTSV CBD, PB2 CBD and eIF4E with the different tracers: **a)** SFTSV CBD with m^{7}GTP-γ-aminophenyl-PEG$_4$-Cy5, **b)** SFTSV CBD with EDA-m^{7}GTP-Cy5, **c)** PB2 CBD wit EDA-m^{7}GTP-Cy5 and **d)** eIF4E with EDA-m^{7}GTP-Cy5. Biological replicates (termed replicate 1-3) were measured in technical triplicates. Fitting and plotting was done in GraphPad Prism 9.5.

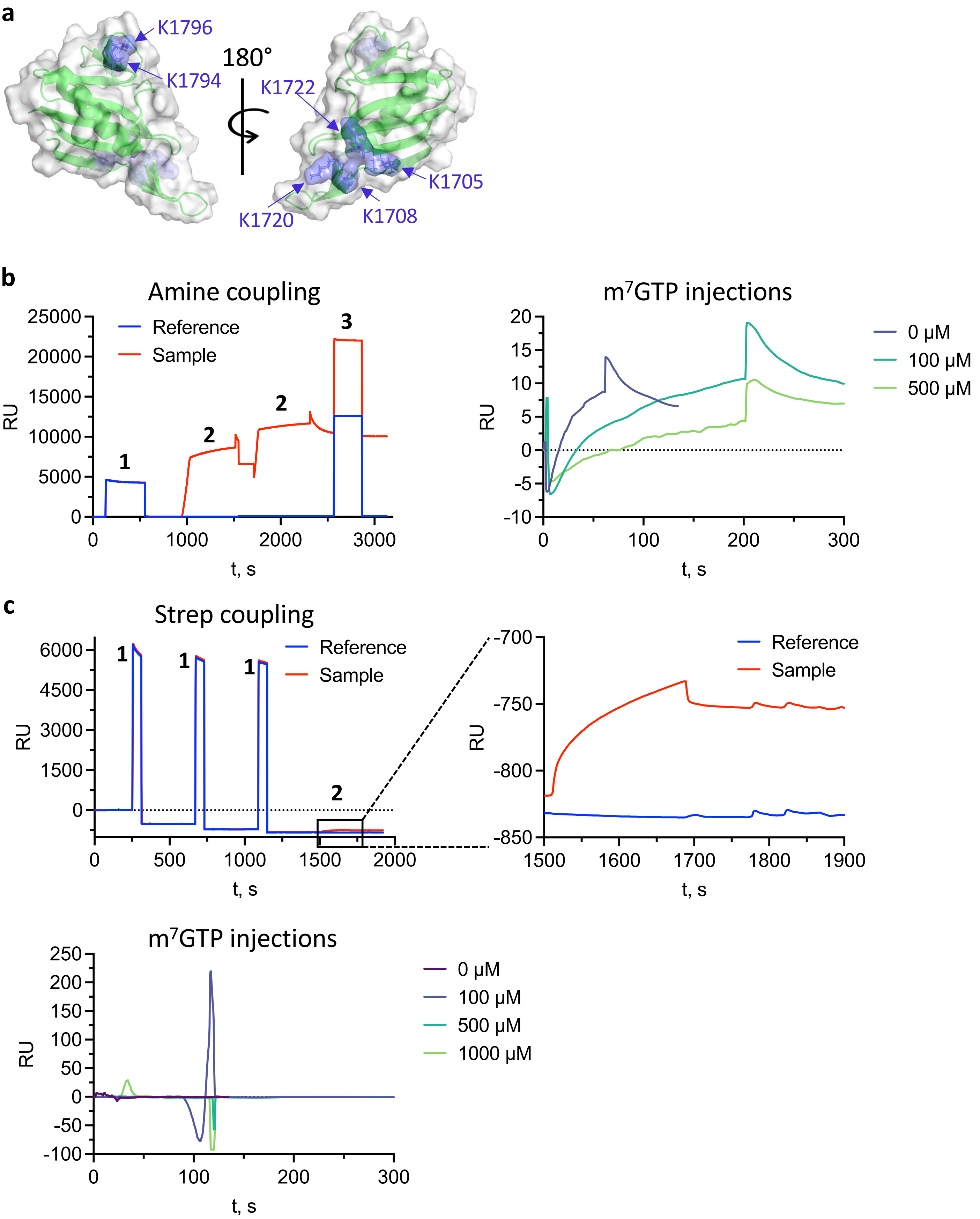

Supplementary Figure 4: Surface plasmon resonance experiments. a) Crystal structures of SFTSV CBD presented as ribbon diagram with the proteins surface in light grey and the lysine residues in blue. The figure was created with PyMOL 2.5. **b)** Immobilization of SFTSV CBD via amine coupling on an amine sensorchip: 1. activation with NHS/EDC, 2. injections of SFTSV CBD, 3. deactivation of unreacted NHS-esters (left). Injection of increasing m^{7}GTP concentrations and on the immobilized SFTSV CBD (right). **c)** Immobilization of SFTSV CBD strep via Strep-tag:NeutrAvidin coupling on a neutravidin coated sensorchip: 1. preparation of sensor surface by injecting NaCL and NaOH, 2. injections of SFTSV CBD strep (upper panel). Injection of increasing m^{7}GTP concentrations on the immobilized SFTSV CBD strep (lower panel). Plots were created in GraphPad Prism 9.5.

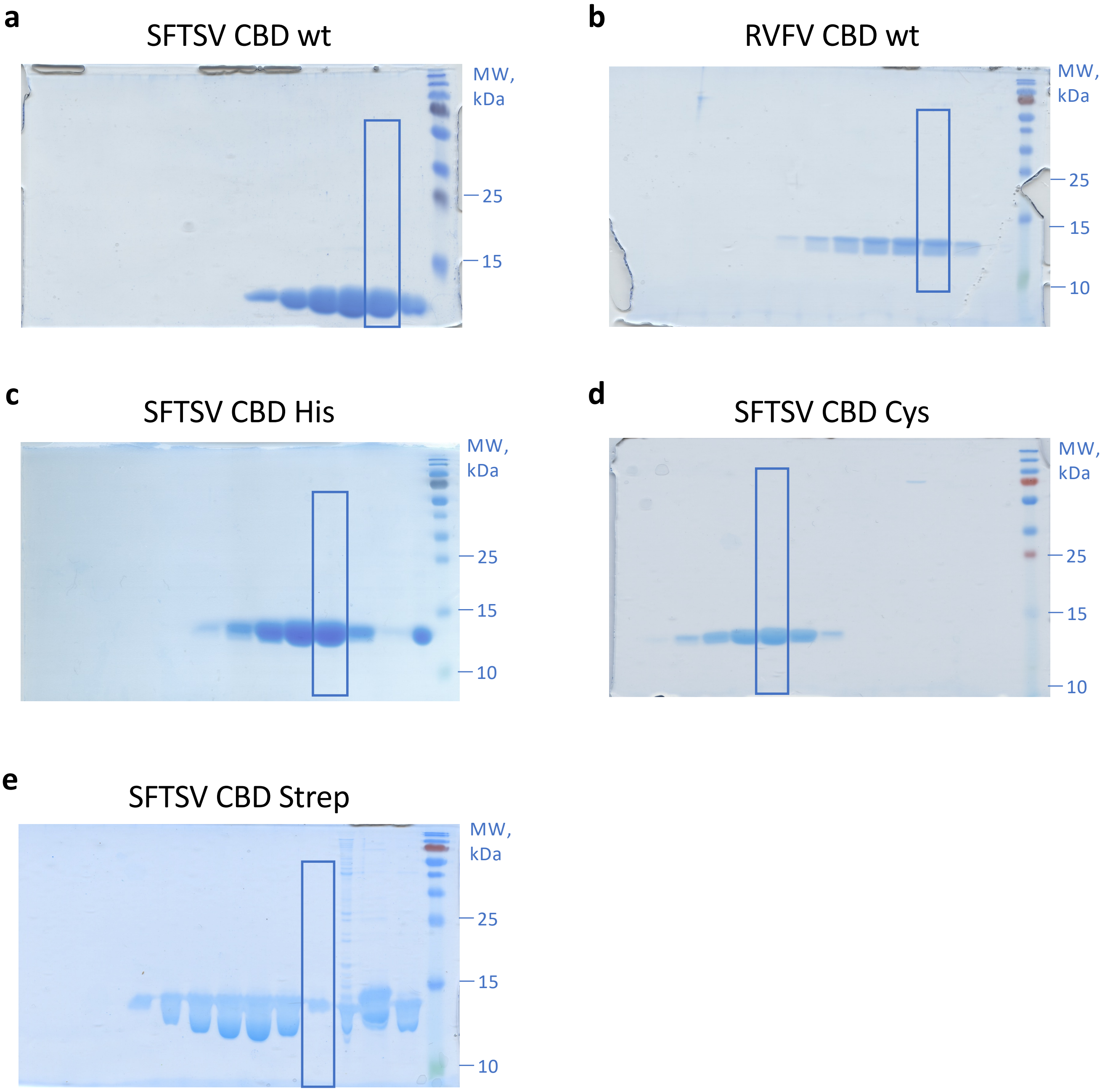

Supplementary Figure 5: Uncropped SDS PAGE gels of CBD mutants. Size exclusion chromatograhy was performed with a Superdex 200 column and protein containing fractions were analyzed via 15% polyacrylamide gels stained with coomassie. The cutouts shown in supplementary figure 1 are marked by boxes. **a)** Wilde type SFTSV CBD. **b)** Wilde type RVFV CBD. **c)** 6xHis tagged SFTSV CBD. **d)** Cys tagged SFTSV CBD. **E)** Strep tagged SFTSV CBD.
